# Epidemiological and molecular characterization of multidrug-resistant *Corynebacterium striatum* in a tertiary hospital in China

**DOI:** 10.1128/spectrum.00280-26

**Published:** 2026-06-30

**Authors:** Xia Li, Hong Li, Chunyan Gao, Ziyang Li, Lili Ding, Yu Wang, Yaping Han, Chenrui Hou

**Affiliations:** 1 Shanxi Bethune Hospital, Shanxi Academy of Medical Sciences, Third Hospital of Shanxi Medical University, Tongji Shanxi Hospital 576225, Taiyuan, China; Universita degli Studi dell'Insubria, Varese, Italy

**Keywords:** *Corynebacterium striatum*, bacterial drug resistance, epidemiology, molecular biology, next-generation sequencing

## Abstract

*Corynebacterium striatum* has emerged as an important opportunistic nosocomial infection; however, epidemiological and antimicrobial resistance data from China remain limited. This study explored the epidemiological distribution, antimicrobial susceptibility profiles, resistance genes, virulence genes, plasmid characteristics, and phylogenetic relationships of clinical *C. striatum* isolates from a tertiary hospital in northern China. A retrospective study was conducted on *C. striatum* isolates recovered between February 2019 and December 2021 from patients with respiratory tract infections, urinary tract infections, catheter-related infections, or sterile-sites infections in which *C. striatum* was the sole pathogen. Antimicrobial susceptibility testing was performed using the E-test method according to CLSI M45 guidelines. Next-generation sequencing was performed for 14 clinical isolates, followed by hybrid assembly, resistance and virulence gene annotation using the CARD and VFDB databases, and single nucleotide polymorphism (SNP) phylogenetic analysis. Plasmid analysis was performed on four bloodstream isolates using Oxford Nanopore sequencing. A total of 312 clinical isolates were identified. All isolates were resistant to penicillin, meropenem, ceftriaxone, and ciprofloxacin but remained susceptible to vancomycin and linezolid. High resistance rates were also observed for tetracycline (69.87%), clindamycin (78.85%), gentamicin (44.23%), and erythromycin (79.17%). Genomic analysis identified 11 antimicrobial resistance genes, including the *erm(X*) gene, which was detected in all clinical isolates. In addition, resistance plasmids were identified, suggesting a potential role in the dissemination of antibiotic resistance. Multidrug-resistant *C. striatum* is prevalent in this hospital setting. Continuous surveillance is warranted, and vancomycin and linezolid remain reliable therapeutic options.

**IMPORTANCE:**

This study addresses a critical gap in the understanding of the emerging hospital pathogen *Corynebacterium striatum* in China. The findings demonstrate that multidrug-resistant strains are already widespread in the clinical setting, with nearly all isolates showing resistance to multiple first-line antibiotics. The identification of resistance genes on mobile plasmids is particularly significant, as it suggests the potential for rapid dissemination to other bacteria. These findings are important for increasing awareness among clinicians and hospitals regarding this underrecognized threat. They also underscore the need for routine testing for accurate identification of this bacterium, support the preferential use of vancomycin or linezolid for treatment, and provide evidence for strengthening infection control measures to prevent outbreaks.

## INTRODUCTION

*Corynebacterium striatum* is a Gram-positive bacillus that commonly colonizes human skin and mucosal surfaces. In recent years, it has been increasingly recognized as a clinically relevant opportunistic pathogen, particularly in hospitalized patients undergoing invasive procedures or prolonged antimicrobial exposure (1, 2). These infections are often associated with patient-specific risk factors and healthcare-related exposures. Notably, the emergence of multidrug-resistant (MDR) *C. striatum* clinical isolates has created substantial therapeutic challenges by limiting treatment options and facilitating bacterial dissemination within hospital environments, thereby posing a significant infection control concern (3). Increasing resistance to β -lactams, macrolides, aminoglycosides, and quinolones has become a major clinical concern (4).

Severe invasive infections caused by this pathogen have been reported in both immunocompetent and immunosuppressed individuals, involving the upper and lower respiratory tract, bloodstream, central nervous system (meningitis), heart (endocarditis), and bones (osteomyelitis) (5). Immunosuppressed patients, including those with neutropenia and acquired immunodeficiency syndrome (AIDS), are particularly susceptible to *C. striatum* infections (6).

Mobile genetic elements, such as plasmids, transposons, and insertion sequences, play a crucial role in the dissemination of resistance genes. Chromosomal mutations and horizontal gene transfer are the primary drivers of antimicrobial resistance (AMR) in *C. striatum*. For example, the *erm(X*) gene, located on transposon Tn5432, has been implicated in resistance to macrolides, lincosamides, and streptogramins (7). Genetic mutations may also occur, such as mutations in the *gyrA* gene that confer resistance to fluoroquinolones (8). Whole-genome sequencing (WGS) has provided deeper insights into the genetic characteristics of *C. striatum*, thereby enhancing the understanding of its resistance mechanisms and evolutionary pathways (9). A previous study conducted in three tertiary hospitals in China investigated quinolone resistance mechanisms and the epidemiological characteristics of *C. striatum*, identifying novel *gyrA* mutations and providing insight into its resistance patterns and clonal diversity (10).

Despite the growing recognition of *C. striatum* as a clinically significant pathogen, important gaps remain in the understanding of its epidemiology and molecular characteristics. Reported antibiotic resistance rates vary widely across studies, complicating clinical decision-making and highlighting the need for further investigation. In particular, data on the epidemiological trends and molecular mechanisms of resistance in *C. striatum* in China remain limited. Therefore, the present study aimed to investigate the epidemiological distribution, antibiotic susceptibility testing (AST) profiles, and resistance phenotypes of clinical *C. striatum* isolates collected from a tertiary hospital in Taiyuan, China. Furthermore, we aimed to identify antimicrobial resistance genes and virulence genes, characterize plasmid structures, and analyze the phylogenetic relationships and evolutionary characteristics of bloodstream isolates using whole-genome sequencing. The ultimate goal was to clarify the molecular mechanisms underlying multidrug resistance and pathogenicity, provide evidence for clinical antimicrobial therapy, and support the optimization of infection prevention and control strategies in healthcare settings.

## RESULTS

### Sample collection

**Fig 1:**
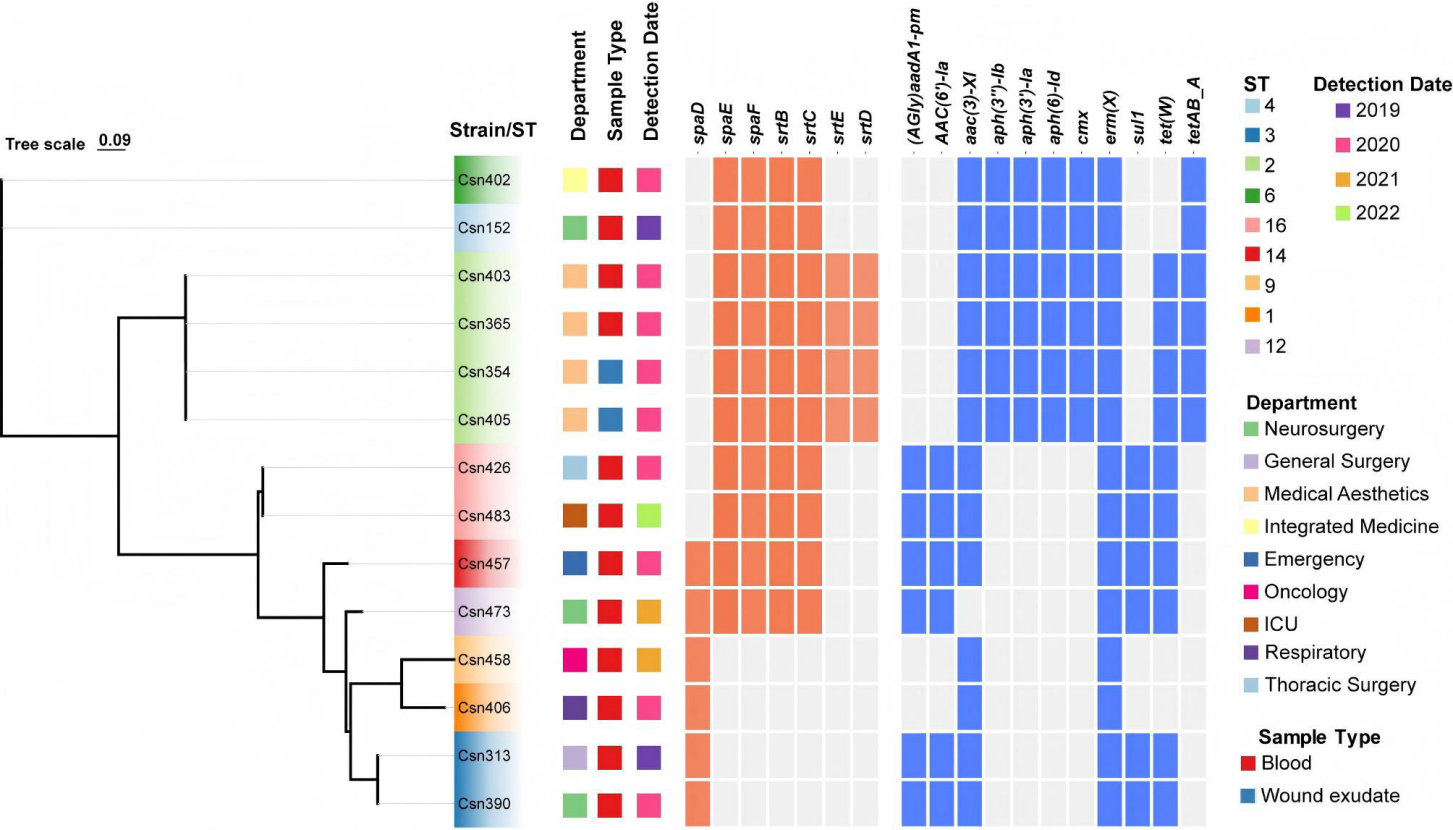
Phylogenetic and phenotypic characterization of clinical *Corynebacterium striatum* isolates from a tertiary hospital in China. Maximum-likelihood phylogenetic tree constructed from core genome single-nucleotide polymorphisms (SNPs) of 14 clinical *C. striatum* isolates (12 bloodstream, 2 wound exudates), with a tree scale of 0.09 nucleotide substitutions per site. Isolate names and assigned sequence types (STs) are indicated at the branch tips. Adjacent color-coded annotation panels denote key metadata for each isolate: department (Neurosurgery, General Surgery, Medical Esthetics, Integrated Medicine, Emergency, Oncology, ICU, Respiratory, Thoracic Surgery), sample type (blood, wound exudate), and detection date (2019–2022). The heatmap on the right displays the presence (filled squares) and absence (empty squares) of clinically relevant antimicrobial resistance genes, including *erm(X)*, *aac(3')-XI*, *tet(W)*, *tetAB_A*, *aadA1-pm*, *aac(6')-Ia*, *aph(3')-Ia*, *aph(3')-Ib*, *aph(6')-Id*, *cmx*, and *sul1.* These genes are associated with resistance to macrolides, tetracyclines, aminoglycosides, chloramphenicol, or sulfonamides, respectively.

A total of 312 *C. striatum* isolates were obtained from patients diagnosed with *C. striatum* infections. The majority of patients (76.60%) were between 50 and 80 years old. The most common source of isolation was sputum samples (191/312, 61.22%), followed by bronchial aspirates (27/312, 8.65%), urine (23/312, 7.37%), wound exudates (17/312, 5.45%), and blood (12/312, 3.85%). These isolates were collected from different departments, with the Department of Neurosurgery contributing the highest proportion (107/312, 34.29%) (Table 1). The first bloodstream infection caused by *C. striatum* in this study was identified in July 2019, with two cases detected that year, followed by seven cases in 2020, two cases in 2021, and one case in the first quarter of 2022. These infections were distributed across multiple departments, including Neurosurgery (3/12), Medical Esthetics (2/12), General Surgery (1/12), Comprehensive Internal Medicine (1/12), Emergency Department (1/12), Oncology (1/12), Intensive Care Medicine (1/12), Pulmonology (1/12), and Thoracic Surgery (1/12) (Fig. 1).

**TABLE 1:** Demographic and clinical features of patients^*a*^

Clinical characteristics	*n* (%)
Age	
≤50	21 (6.73%)
51–79	239 (76.60%)
≥80	52 (16.67%)
Gender	
Female	81 (25.96%)
Male	231 (74.04%)
Department	
Neurosurgery	107 (34.29%)
Neurology	55 (17.63%)
Rehabilitation medicine	32 (10.26%)
Respiratory and critical care	29 (9.29%)
General practice	25 (8.01%)
Sample type	
Sputum	191 (61.22%)
Bronchialaspiration sample	27 (8.65%)
Urine	23 (7.37%)
Wound exudate	17 (5.45%)
Blood	12 (3.85%)
Underlying conditions	
Intracerebral hemorrhage	106 (33.97%)
Cerebral infarction	71 (22.76%)
Pulmonary infection Consciousness disorder	38 (12.18%) 29 (9.29%)
Cancer	15 (4.81%)

^*a*^ A given patient could fit into more than one category.

In the present study, all patients from whom *C. striatum* was isolated from sputum presented with symptoms of lung infection. Clinicians suspected pulmonary infection and submitted sputum specimens for culture. The epithelial cell counts in smears of these sputum samples collected during the same period were all <10 per low-power microscopic field.

### Identification of *C. striatum* isolates

MALDI-TOF mass spectrometry was used to reconfirm bacterial strain identification. First, a standard solvent was prepared by mixing acetonitrile, deionized water, and trifluoroacetic acid. The α-CHCA matrix solution was then prepared using this solvent and stored in the dark at room temperature. For sample preparation, single bacterial colonies were applied to target plate wells and overlaid with matrix solution, followed by air drying to form crystal films for detection. BTS was used for instrument calibration with the following parameter settings: 40 shots, 60 Hz frequency, and six overlaps, resulting in a total of 240 shots. Calibration was considered acceptable only when eight reference protein peaks matched within an error range of ≤±300 ppm. Sample spectra were acquired using the same method as that used for calibration and subsequently saved. The data were imported into the Biotyper software and compared with the Bruker 5989 database. The reconfirmation identification accuracy for all 312 Corynebacterium striatum isolates was 100%, with all identification scores exceeding 1.5.

### Antimicrobial susceptibility testing

Antimicrobial susceptibility testing of the *C. striatum* isolates revealed a consistent resistance pattern across the clinical isolates. All isolates demonstrated resistance to penicillin, meropenem, ceftriaxone, and ciprofloxacin while remaining 100% susceptible to vancomycin, linezolid, and daptomycin. Notably, a high significant proportion of total isolates exhibited resistance to clindamycin (78.85%), erythromycin (79.17%), tetracycline (69.87%), gentamicin (44.23%), chloramphenicol (50.00%), trimethoprim-sulfamethoxazole (30.13%), and rifampicin (4.49%). However, all 12 bloodstream isolates were susceptible to rifampicin and completely resistant to erythromycin and clindamycin. The minimum inhibitory concentration (MIC) values and susceptibility profiles for each antimicrobial agent indicated that vancomycin, linezolid, or daptomycin should be considered first-line therapeutic options, whereas β-lactams, fluoroquinolones, macrolides, lincosamides, and tetracyclines were ineffective (Table 2).

**TABLE 2:** Minimum inhibitory concentration distributions and CLSI interpretations for *Corynebacterium striatum* clinical isolates

Antibiotics	MIC^*a*^ (µg/mL) interpretive criteria			Bacterial clinical isolates of different minimum inhibitory concentration (µg/mL) n^*b*^/Interpretation result											Susceptible clinical isolates (%)	
	S	I	R	0.25	0.5	1	2	4	8	16	32	64	128	256	Blood	Total
Penicillin	≤0.12	0.25–2	≥4								312/R				0.00	0.00
Meropenem	≤0.25	0.5	≥1								312/R				0.00	0.00
Ceftriaxone	≤1	2	≥4								312/R				0.00	0.00
Tetracycline	≤4	8	≥16		45/S	17/S	15/S		17/I	102/R	45/R	71/R			25.00	24.68
Ciprofloxacin	≤1	2	≥4						71/R	155/R	86/R				0.00	0.00
Clindamycin	≤0.5	1–2	≥4	56/S			10/I	15/R		160/R	36/R	35/R			0.00	17.95
Erythromycin	≤0.5	1	≥2	61/S		4/I				53/R	142/R	52R			0.00	19.55
Gentamicin	≤4	8	≥16	80/S	53/S		15/S	5/S	21/I	77/R		46/R	15/R		41.67	49.04
Rifampicin	≤1	2	≥4	281/S	17/S			9/R	5/R						100.00	95.51
Vancomycin	≤2	–	–	310/S	24/S										100.00	100.00
Linezolid	≤2	–	–	295/S	17/S										100.00	100.00
Daptomycin	≤1	–	–	302/S	10/S										100.00	100.00
Chloramphenicol^*c*^	≤8	16	≥32	15/S	78/S	47/S				16/I		16/R		140/R	50.00	50.00
Trimethoprim-sulfamethoxazole	≤2	–	≥4	47/S	62/S	109/S		16/R			78/R				66.67	69.87

^*a*^ MIC breakpoint based on the CLSI M45-A3 standard (S represents susceptible, I represents intermediate, R represents resistant).

^*b*^ “n” represents the number of cases.

^*c*^ MIC breakpoint based on the CLSI M100 standard for *Staphylococcus* spp.

### Validation of antimicrobial resistance genes

To further investigate the resistance mechanisms of these 14 clinical *C. striatum* isolates, antimicrobial resistance gene analysis was performed. The analysis identified multiple resistance genes associated with resistance to β-lactams, macrolides, tetracyclines, aminoglycosides, and sulfonamides. A total of 11 antimicrobial resistance genes were identified across the *C. striatum* genomes. Specifically, the following genes were detected: *aadA1-pm*, *aac(6')-Ia*, *aac(3)-XI*, *aph(3'')-Ib*, *aph(3')-Ia*, *aph(6)-Id*, *cmx*, *erm(X)*, *sul1*, *tet(W)*, and *tetAB_A*. All 14 isolates carried the *erm(X*) gene, which is associated with macrolide resistance, whereas 13 isolates carried the *aac(3)-XI* gene. In addition, *aadA1-pm* and *aac(6’)-Ia* were identified in six isolates, while *aph(3”)-Ib*, *aph(3’)-Ia*, and *aph(6)-Id* were detected in another six isolates; all of these genes are associated with aminoglycoside resistance. The *cmx* gene, linked to chloramphenicol resistance, and the *tetAB_A* gene, associated with tetracycline and β-lactam resistance, were each detected in six isolates. The sul1 gene, responsible for sulfonamide resistance, was present in six isolates, whereas the *tet(W*) gene, which confers tetracycline resistance, was identified in 10 isolates. Notably, 12 isolates simultaneously carried more than five resistance genes.

Several resistance genes, including *RbpA* (associated with rifampicin resistance), *adeH* (associated with ciprofloxacin resistance), and *TEM-210* (associated with β-lactam resistance), were detected; however, their identity scores were below the identification threshold (80%). No resistance genes associated with vancomycin, linezolid, or daptomycin resistance were detected, which was consistent with the observed full susceptibility. The detected resistance genes suggested potential resistance mechanisms, although they did not always correspond to the expressed phenotypes

### Analysis of virulence and sequence types in *C. striatum*

Analysis of isolates from bloodstream and wound infections revealed four distinct combinations of virulence genes, namely (i) *spaD+*, (ii) *spaDEF+/srtBC+*, (iii) *spaEF+/srtBC+*, and (iv) *spaEF+/srtBCDE+*. The combinations *spaDEF*+/*srtBC*+ was identified in two isolates, whereas the other combinations (*spaD*+, *spaEF*+*/srtBC*+ and *spaEF*+*/srtBCDE*+) were identified in four isolates each. The virulence profiles of the two strains isolated from wound exudates were identical to those of bloodstream isolates obtained from blood within the same department.

Concurrently, NGS, BLAST, and phylogenetic analyses were performed on two *C. striatum* isolates recovered from wound secretions. Interestingly, the virulence gene and antimicrobial resistance gene profiles of the two wound exudate isolates were identical to those of bloodstream isolates obtained from the same department. Among the 12 bloodstream infection isolates, multiple sequence types (STs) were identified, including ST1, ST2, ST3, ST4, ST6, ST9, ST12, ST14, and ST16. Two isolates each belonged to ST2, ST3, and ST16, whereas the remaining STs (ST1, ST4, ST6, ST9, ST12, and ST14) were represented by a single isolate (Fig. 1).

### Global traceability and evolutionary analysis of *C. striatum*

**Fig 2:**
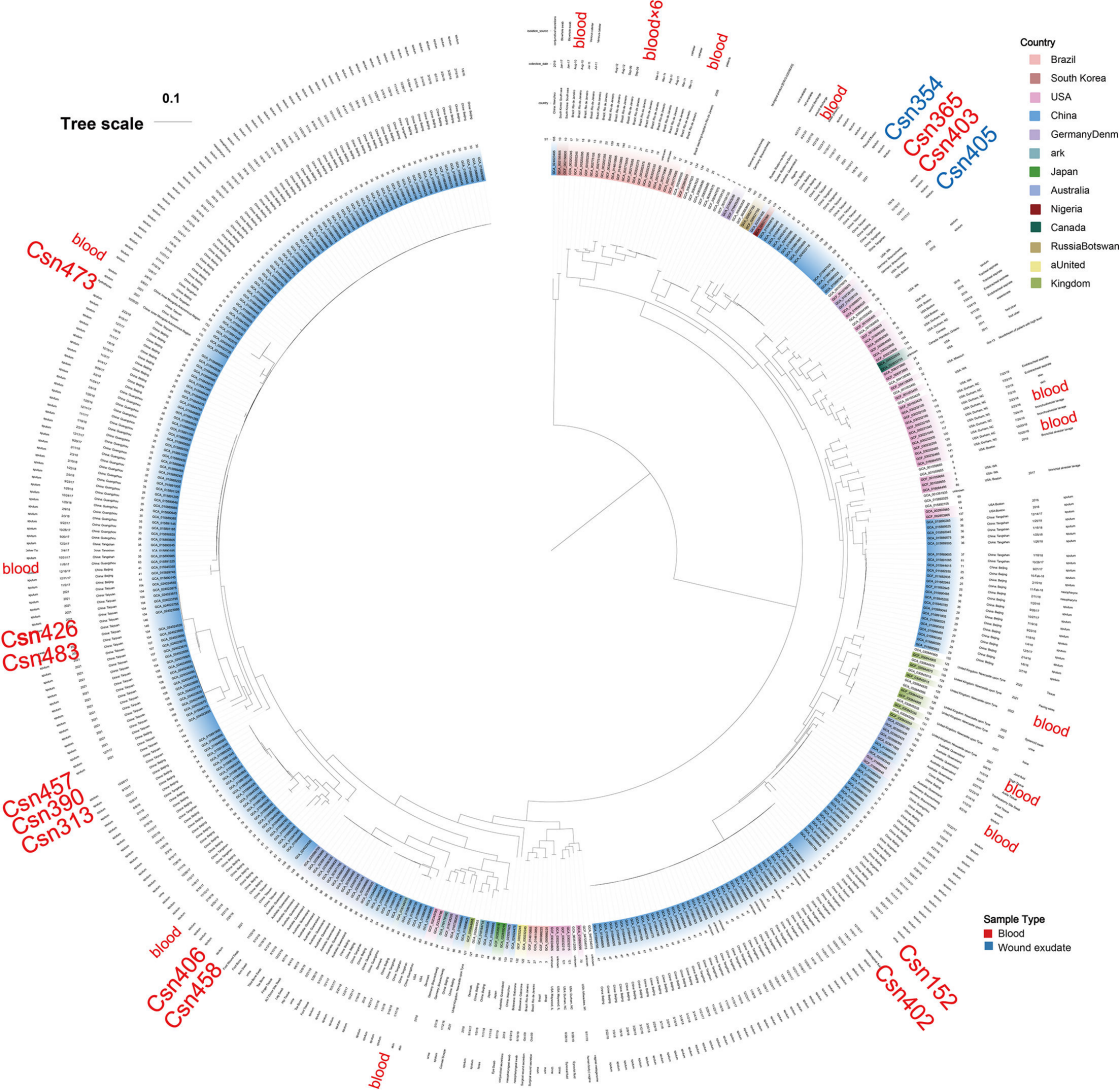
Global core-genome SNP-based phylogenetic analysis of *Corynebacterium striatum* clinical isolates. Maximum-likelihood phylogenetic tree constructed from core genome single-nucleotide polymorphisms (SNPs) of *Corynebacterium striatum* clinical isolates, including study isolates (labeled with *Csn* prefix) and publicly available global *C. striatum* strains from multiple countries (Brazil, South Korea, USA, China, Germany, Denmark, Japan, Australia, Nigeria, Canada, Russia, Botswana, United Kingdom). The tree scale of 0.1 indicates the number of nucleotide substitutions per site. Isolate metadata are annotated with color-coded labels for isolation source/country, sample type (blood, wound exudate), and collection date for the study isolates; key labels for the study strains include *collection_date* and *isolation_source* (blood) for contextualization. All study isolates included in this analysis were recovered from blood or wound exudate specimens, with bloodstream isolates representing the primary cohort.

Phylogenetic analysis based on single-nucleotide polymorphisms demonstrated that the 12 bloodstream *C. striatum* isolates and two wound isolates clustered closely with previously reported clinical infection *C. striatum* isolates from China. Comparative genomic analysis indicated that these isolates were genetically related to strains originating from Beijing, Tangshan, Inner Mongolia, and Taiyuan, whereas isolate Csn458 showed the closest genetic relatedness to a strain reported from Queensland, Australia (Fig. 2).

### Plasmid analysis of four *C. striatum* clinical isolates

**Fig 3:**
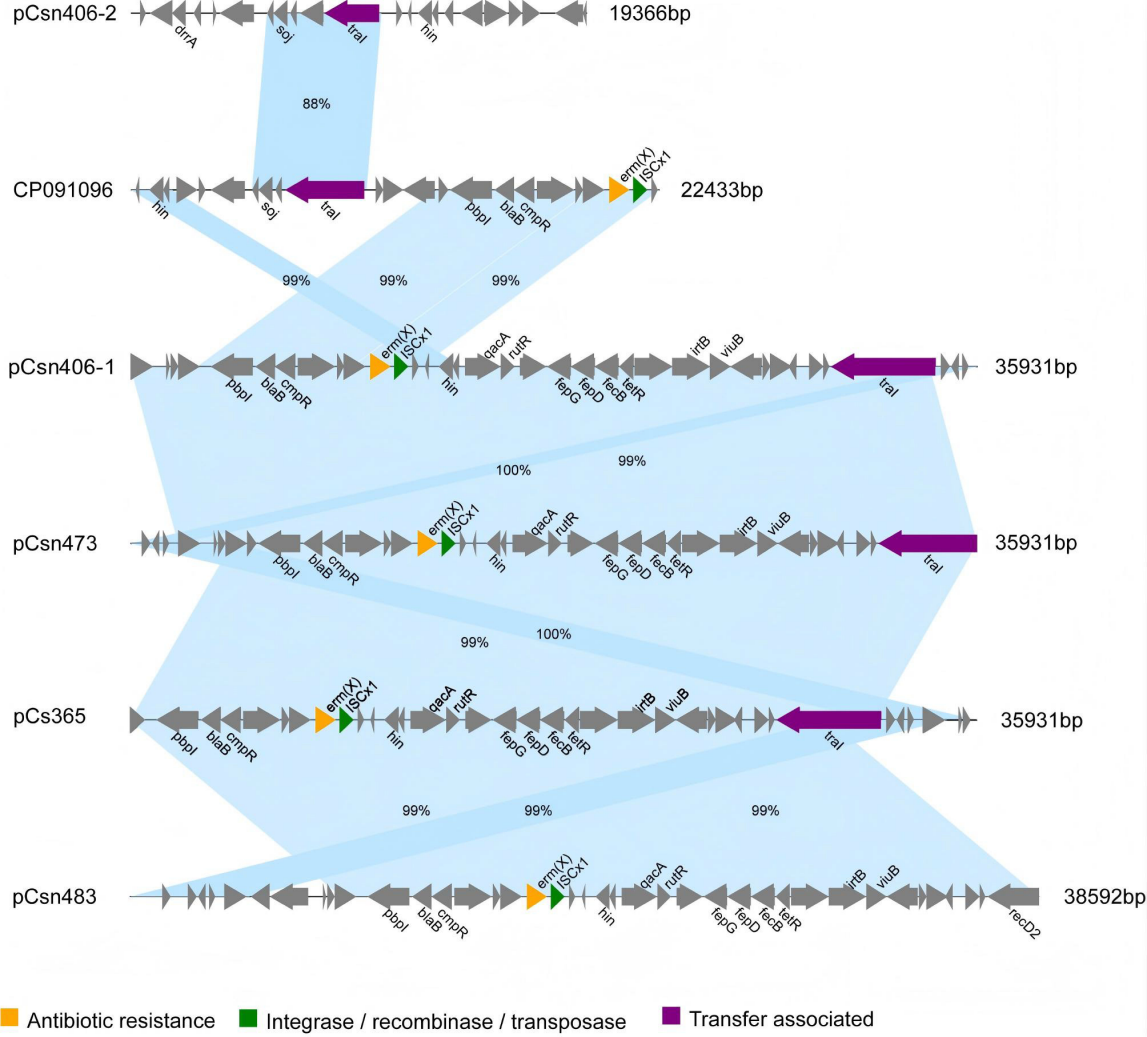
Comparative linear alignment of plasmids from clinical *Corynebacterium striatum* isolates. Linear genomic alignment generated using Easyfig (v2.2.5) to visualize synteny and gene content across six plasmids (pCsn406-2, CP091096, pCsn406-1, pCsn473, pCsn365, pCsn483) from clinical *Corynebacterium striatum* isolates. Light blue shaded regions indicate segments with ≥99% nucleotide sequence identity between adjacent plasmids (88% identity is shown for the *traI* region between pCsn406-2 and CP091096). Arrows represent open reading frames (ORFs), with functional categories color-coded: orange for antibiotic resistance genes (e.g., *erm(X*)), green for integrase/recombinase/transposase genes (e.g., *ISCx1*), and **purple** for transfer-associated genes (e.g., *traI*). Key shared loci include *blaB*, *cmpR*, *qacA*, and type IV secretion system components (*ihtB*, *viuB*), highlighting conserved backbone and accessory gene regions across these plasmids. The scale bar indicates nucleotide position.

Whole-genome sequencing of four clinical C. striatum isolates recovered from bloodstream infections revealed that isolate Csn406 carried two plasmids, pCsn406-1 and pCsn406-2, with sizes of 359,311 and 19,366 bp, respectively. Notably, pCsn406-1 was identified as a resistance plasmid carrying the *erm(X*) erythromycin resistance gene. Clinical isolates Csn365, Csn473, and Csn483 each carried a single plasmid harboring resistance genes (Fig. 3).

Detailed analysis revealed that plasmids pCsn406-1, pCsn365, and pCsn473 were identical in size and exhibited 100% sequence identity, indicating that they were essentially the same plasmid. The pCsn483 plasmid, with a size of 385,921 bp, shared 86% sequence similarity with the aforementioned plasmids.

**Fig 4:**
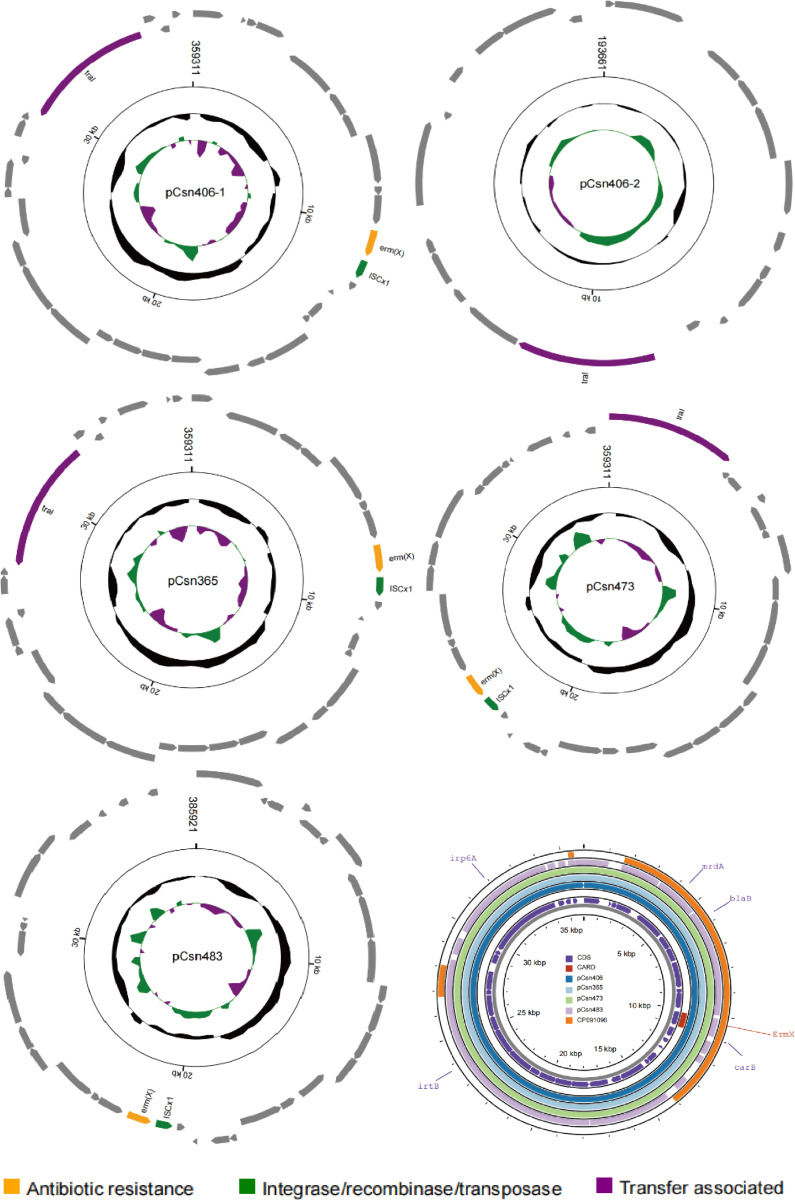
Comparative linear alignment of resistance plasmids from clinical *Corynebacterium striatum* isolates. Linear genomic alignment of plasmids from four *Corynebacterium striatum* bloodstream isolates (pCsn406-1, 359311 bp; pCsn406-2, 19366 bp; pCsn365, 359311 bp; pCsn473, 359311 bp; pCsn483, 385921 bp) and the reference *Corynebacterium diphtheriae* plasmid CP091096. The scale bar indicates nucleotide position in kilobase pairs (kbp). Open reading frames (ORFs) are color-coded by functional category according to the legend: orange for antibiotic resistance genes (including the conserved *erm(X*) macrolide resistance gene), green for integrase/recombinase/transposase genes, and purple for transfer-associated genes. Key resistance and accessory genes (e.g., *carB*) are labeled; pCsn365 and pCsn473 share identical size and sequence, whereas pCsn483 shows high homology to the reference plasmid CP091096 and harbors a conserved resistance gene backbone shared with the other clinical plasmids.

BLAST comparison revealed that pCsn483 showed minimal differences from the classical *C. diphtheriae* plasmid CD1009 (NC_CP091096.1), with a 99% sequence similarity (Fig. 4). In addition, comparison against the coding DNA sequence (CDS) database identified multiple β-lactam resistance genes, including mrdA, blaB, and carB (11, 12). Moreover, two virulence genes, irp6A and irtB (13, 14), were identified in *C. striatum* for the first time through genomic analysis. The irp6A gene is involved in the siderophore-dependent iron uptake system (13), whereas irtB encodes an ABC transporter (14). The virulence functions of these genes were inferred through bioinformatic analysis, and no functional validation was performed. All whole-genome sequencing data from the four bloodstream *C. striatum* isolates generated by Nanopore sequencing were deposited in a public database under BioProject ID PRJNA1371382.

## DISCUSSION

The high prevalence of multidrug-resistant *C. striatum* clinical isolates poses a serious challenge to clinical treatment. Resistance plasmids play a crucial role in the dissemination of antibiotic resistance genes. For example, plasmid-mediated horizontal gene transfer enables the spread of the *erm(X*) gene, which confers resistance to macrolides, lincosamides, and streptogramins, to spread among clinical isolates (15). This not only limits therapeutic options for patients infected with *C. striatum* but also increases the risk of hospital-acquired infections. Clinically, appropriate antibiotics should be selected based on susceptibility testing results to improve treatment efficacy. Moreover, understanding the resistance mechanisms and genetic characteristics of these clinical isolates may facilitate the development of targeted infection control strategies, such as strengthening environmental monitoring and improving hand-hygiene compliance, to prevent the dissemination of resistant isolates in hospital settings (7).

From an epidemiological perspective, *C. striatum* has been increasingly associated with human infections since its first report in 1980 (16). It can cause severe local or systemic infections in high-risk patients. Multidrug-resistant *C. striatum* clinical isolates have also been reported to cause hospital-acquired infection outbreaks worldwide. Our findings suggest that, in the hospital setting, specific patient groups—including males, individuals older than 50 years, patients in the neurosurgery department, and those with certain underlying diseases—are at increased risk of *C. striatum* infection. One possible explanation is that neurosurgery patients often undergo invasive procedures that disrupt normal host defense mechanisms, thereby increasing susceptibility to *C. striatum* infection. In addition, the high detection rate in the respiratory medicine and rehabilitation departments may be attributed to both interdisciplinary referrals and the clinical characteristics of patients in these departments (17). Many patients in respiratory medicine have underlying respiratory diseases that compromise immune function, whereas patients in rehabilitation departments may have weakened physical conditions due to prolonged illness, both of which create favorable conditions for the colonization and infection of *C. striatum* (18). Notably, a study from Japan also highlighted the predominance of *C. striatum* infections among male patients and individuals with underlying cerebrovascular diseases (2). The high detection rate in respiratory specimens further reflects global trends in which colonization precedes infection in immunocompromised hosts, particularly those with chronic lung diseases or prolonged hospital stays (19). These consistent epidemiological patterns underscore the need for standardized surveillance protocols targeting high-risk populations identified in our analysis to mitigate the growing threat of *C. striatum* as an emerging nosocomial pathogen (20).

*C. striatum* has increasingly been implicated in bloodstream infections, particularly in hospitalized or immunocompromised patients, including those with central venous catheters or extracorporeal membrane oxygenation (ECMO) systems. These infections are frequently catheter-related, and the isolates are capable of forming biofilms that create therapeutic challenges by impairing antibiotic penetration. Reported invasive manifestations include bacteremia, endocarditis, meningitis, pneumonia, osteomyelitis, and peritoneal or device-associated infections. In bloodstream infections, vancomycin remains the most effective therapeutic option, although resistance to most oral antibiotics, including penicillin, clindamycin, tetracycline, and fluoroquinolones, is common (21–23). WGS and pan-genomic analyses have identified a wide range of antimicrobial resistance determinants and mobile genetic elements in *C. striatum*. The pTP10 plasmid harbors multiple antibiotic resistance genes, and isolates frequently possess the aminoglycoside resistance genes *aph(3’)-Ic*, *aph(3”)-Ib*, and *aph(6)-Id*. Analysis of a multidrug-resistant bloodstream isolate from Brazil also identified CRISPR-Cas systems and SpaDEF clusters, indicating adaptive immunity and virulence-associated characteristics. Comparative genomic analyses have demonstrated widespread clonality in healthcare-associated outbreaks, suggesting persistence and nosocomial transmission facilitated by biofilm formation and environmental survival (20, 23, 24). Non-bloodstream infections caused by *C. striatum* include exit-site and tunnel infections in dialysis patients, as well as device-associated infections, such as prosthetic joint or cardiac device infections. These infections highlight the organism’s ability to colonize and persist on abiotic surfaces. The clinical impact is further amplified by the requirement for prolonged intravenous antimicrobial therapy and the potential for epidemic spread within healthcare environments, underscoring its role as an underrecognized nosocomial pathogen (1, 22, 25). Therefore, NGS- and WGS-based studies have transformed the understanding of *C. striatum* from a benign commensal organism into a genetically adaptable multidrug-resistant pathogen implicated in invasive and bloodstream infections.

One of the most serious challenges in the treatment of infections caused by *C. striatum* is the emergence of multidrug-resistant clinical isolates. In this study, all 312 collected isolates were multidrug-resistant (16), consistent with the global trend of increasing antimicrobial resistance in *C. striatum* infections. This finding is further supported by another study that identified 54 multidrug-resistant *C. striatum* clinical isolates from a local hospital, emphasizing the extent of this problem (16). This multidrug resistance substantially complicates treatment decisions. Therefore, the selection of appropriate antibiotics is critical, and antimicrobial susceptibility testing results should guide the treatment of *C. striatum* infections. In our study, all clinical isolates were susceptible to vancomycin, linezolid, and daptomycin, which is consistent with the findings reported by Asgin et al. (26). In contrast, all *C*. *striatum* clinical isolates were resistant to penicillin, ceftriaxone, and ciprofloxacin. The *bla* genes encoding class A β-lactamases are associated with penicillin resistance, whereas the *ampC* genes encoding class C β-lactamases are associated with ceftriaxone resistance. Quinolone resistance is associated with point mutations in the DNA gyrase A subunit encoded by the gyrA gene (26). Nevertheless, Ikegaki et al. (27) reported 11 isolates from patients that were initially susceptible to daptomycin but became nonsusceptible during treatment, highlighting the potential for resistance development.

Neemuchwala et al. (28) reported high resistance rates among 931 C. striatum isolates collected in Canada between 2011 and 2016, with resistance rates of 99.9% to penicillin and 95.4% to ciprofloxacin. In contrast, Alibi et al. (6) reported lower resistance rates among 63 *C. striatum* isolates collected between 2011 and 2014, with resistance rates of 82.5% to penicillin and 36.5% to ciprofloxacin. These differences may be attributed to regional variations in antibiotic resistance profiles and differences in collection periods. In regions with intensive use of specific antibiotics, bacteria are exposed to greater selective pressure, leading to a higher prevalence of resistant clinical isolates (29, 30).

The most common mechanism of *C*. *striatum* resistance to aminoglycoside antibiotics is the production of enzymes that modify the antibiotic molecules, thereby conferring resistance (31). In the present study, the resistance rate to gentamicin was 44.23% (138/312), compared with 7.2% reported by Neemuchwala et al. (28) and 6.3% reported by Alibi et al. (6). This substantial difference further highlights the influence of local factors on antibiotic resistance patterns. In our study, erythromycin and clindamycin exhibited high resistance rates of 79.17% (247/312) and 78.85% (246/312), respectively. Macrolide resistance is primarily associated with methylation of the antibiotic binding site by methylase enzymes, which are mainly encoded by the *erm(X*) gene (32). The *tetA* and *tetB* genes confer tetracycline and β-lactam resistance in *C. striatum* through active efflux pump mechanisms. The *cmx* gene is associated with chloramphenicol resistance and also encodes an efflux pump. The *tet(W*) gene encodes a ribosomal protection protein that modifies the ribosome through non-covalent interactions, thereby preventing tetracycline-mediated inhibition of protein synthesis (33).

Among the isolates, the *erm(X*) gene was detected in 14 clinical isolates, whereas the *aac* (3)*-XI* gene was identified in 13 isolates. In addition, 12 isolates carried at least five antibiotic resistance genes, which explains the multidrug resistance observed in these clinical isolates. Virulence gene analysis identified the *spa* and *srt* virulence genes*,* which encode a complete set of pilus proteins and their corresponding sortases. This is the first report in *C. striatum* indicating that these genes encode pili that facilitate bacterial adhesion to and invasion of host cells (34). Pan-genomic analysis further revealed that these pilus genes co-occurred with iron-uptake systems such as irp6A/B/C in 100% of clinical isolates, suggesting coordinated mechanisms of adhesion and nutrient acquisition (24). These consistent findings underscore the role of horizontally transferred genetic elements in shaping the pathogenic potential of *C*. *striatum*.

Whole-genome sequencing results for four clinical C. striatum isolates revealed that all isolates carried antibiotic resistance plasmids. Comparison with the CARD database identified the presence of the erythromycin resistance gene *erm(X*). This gene confers resistance to lincosamides and macrolides, including clindamycin and erythromycin. Clinically important *Corynebacterium* species exhibit widespread resistance to lincosamides and macrolides, which is associated with the *erm(X*) gene carried by transposon Tn5432 (7). The first region of the pTP10 plasmid contains transposon Tn5432. The presence of ISCx1 elements adjacent to the *erm(X*) gene has also been reported (35), which is consistent with the findings of the present study. In addition, the β-lactam resistance genes *mrdA*, *blaB*, and *carB* were detected on the plasmid. The *mrdA* gene encodes penicillin-binding protein 2 (PBP2), which is involved in bacterial cell wall synthesis. Mutations or altered expression levels of this gene are associated with β-lactam resistance in bacteria (36). The virulence genes irp6A and *irtB* were also identified. The *irp6A* gene is involved in the siderophore-dependent iron uptake system and has previously been detected in *Corynebacterium diphtheriae*. The protein encoded by *irtB* is part of an ABC transporter, and the function of the *irtB* gene is essential for the replication and survival of *Mycobacterium tuberculosis* because of its role in iron uptake (37). Both genes are important virulence-associated factors, and their presence in *C. striatum* may contribute to its pathogenicity and the severity of infection.

### Limitations

This study has several limitations. Although 312 clinical isolates were collected from multiple departments within a tertiary hospital, the sample may not fully represent *C. striatum* infections in other healthcare settings. The study was limited to patient records from the study hospital; therefore, information regarding prior antibiotic use at local hospitals or clinics was unavailable. In addition, some resistance-associated mutations were not routinely assessed during clinical management and therefore could not be analyzed. Differences in patient populations and hospital practices may also affect the generalizability of the findings, highlighting the need for larger multicenter studies. Furthermore, the relatively short study period limited the assessment of long-term epidemiological and resistance trends. Finally, this study mainly focused on resistance and virulence genes, whereas other important factors, including environmental influences, host immune responses, and biofilm formation, were not investigated and warrant further study.

This study analyzed 312 clinical *C. striatum* isolates collected from a tertiary hospital in Taiyuan, China, and characterized their infection features. *C. striatum* infections were most commonly identified in elderly male patients, particularly those in the neurosurgery department and those with conditions, such as cerebral hemorrhage. All clinical isolates were resistant to penicillin, meropenem, ceftriaxone, and ciprofloxacin but remained susceptible to vancomycin and linezolid. Genomic analysis identified 11 antimicrobial resistance genes, including *erm(X),* in all clinical isolates, but plasmid-mediated resistance and potential horizontal transfer were inferred from only four isolates. These findings have important implications for antibiotic selection and antimicrobial stewardship. Future studies should focus on larger multicenter investigations to further clarify the epidemiology, resistance mechanisms, and the influence of environmental and host factors on *C. striatum*.

## MATERIALS AND METHODS

### Study design

This single-center retrospective study was conducted at a tertiary hospital in China. Data were collected from electronic medical records, microbiological reports, and laboratory databases between February 2019 and December 2021. The study was approved by the institutional review board, and the requirement for individual informed consent was waived due to its retrospective design. This manuscript adheres to the Strengthening the Reporting of Observational Studies in Epidemiology (STROBE) Statement (38).

### Inclusion and exclusion criteria

Patients were included if *C. striatum* was isolated as the sole pathogen from clinical specimens and if clinical manifestations were consistent with respiratory, urinary, bloodstream, or catheter-related infections. Microbiological criteria included respiratory specimens with <10 epithelial cells and ≥25 white blood cells per low-power field (LPF) and Gram-positive rods on direct Gram staining (39), urine specimens with >5 white blood cells per high-power field (HPF) and *C. striatum* growth ≥10⁴ CFU/mL (40); catheter-related infections in which *C. striatum* growth ≥15 CFU per 5 cm of catheter (2); and bloodstream infections where *C. striatum* was the only identified organism (41). For bloodstream infections, one to three blood culture bottles were collected from each patient, and positive results were obtained within 2 days of sampling. Patients were excluded if they had incomplete clinical data, polymicrobial infections, colonization without infection-related symptoms, prior antimicrobial therapy that could affect culture results, or samples deemed contaminated by laboratory quality control.

### Isolation and Identification of *C. striatum*

During the study period, clinical specimens were routinely cultured on blood agar plates (Antu, Zhengzhou, China) and incubated at 35°C in a 5% CO_2_ incubator for 24 to 48 h. All isolates were identified as *C. striatum* using matrix-assisted laser desorption/ionization time-of-flight mass spectrometry (MALDI-TOF MS, Bruker, Germany) (42, 43). This method uses characteristic mass spectral profiles of microbial proteins to rapidly and accurately identify bacterial species at both the genus and species levels. Identification scores ranged from 0 to 3. Scores <1.7 were considered unreliable, scores between 1.7 and 2.0 were considered acceptable for genus-level identification, and scores >2.0 were considered reliable for species-level identification (44).

### Antimicrobial susceptibility testing

During the study period, antimicrobial susceptibility testing was routinely performed using Mueller-Hinton agar plates (Antu, Zhengzhou, China) and E-test strips (Antu, Zhengzhou, China). The E-test strips included penicillin, meropenem, ceftriaxone, tetracycline, ciprofloxacin, clindamycin, erythromycin, gentamicin, vancomycin, rifampicin, and linezolid at concentrations consistent with CLSI documentation (as indicated in the tables). The plates were incubated at 37°C in an incubator for 24 h. The selection of antimicrobial agents and the interpretation of susceptibility breakpoints followed the guidelines established by the Clinical and Laboratory Standards Institute (CLSI) 2016 document.

### Genome sequencing

Genomic DNA was extracted using the SDS method. The extracted DNA was evaluated by agarose gel electrophoresis and quantified by Qubit 2.0 Fluorometer (Thermo Scientific). Sequencing libraries were constructed using the NEBNext Ultra DNA Library Prep Kit for Illumina (NEB, USA) following the manufacturer’s instructions, and index codes were added to assign sequences to each sample. Briefly, DNA samples were fragmented by sonication to an average size of 350 bp, followed by end repair, A-tailing, and ligation with the full-length adaptor for Illumina adapters, with subsequent PCR amplification. Finally, PCR products were purified using the AMPure XP system, and library fragment size distribution was assessed using an Agilent 2100 Bioanalyzer, while quantification was performed by real-time PCR.

Meanwhile, four isolates underwent third-generation sequencing using Nanopore technology. Libraries for Nanopore sequencing were constructed with an insert size of 10 kb. First, high-molecular-weight DNA fragments were selected using the BluePippin automated nucleic acid size-selection system and subsequently repaired. Barcodes were added using a PCR-free method with the EXP-NBD104 kit from Oxford Nanopore Technologies. The fragment size distribution was then evaluated using an AATI automated capillary electrophoresis instrument, and samples were pooled at equimolar concentrations. Finally, adapters were ligated using the SQK-LSK109 ligation kit to construct the 10 kb library.

The whole genome of *C. striatum* was sequenced using the Nanopore PromethION platform and the Illumina NovaSeq PE150 platform at the Beijing Novogene Bioinformatics Technology Co., Ltd.

### Assembly and annotation

For the complete genome, a hybrid assembly strategy integrating Illumina short reads and Nanopore long reads was employed. Assembly was performed using Unicycler: an initial assembly framework was generated with SPAdes based on short reads, while miniasm and Racon were used to construct long-read bridges and resolve assembly conflicts. Circular structures were validated, replication initiation sites were adjusted based on dnaA and repA, and all gaps were fully closed to generate a complete, gap-free complete genome sequence. For the draft genome, only Illumina short-read data were used. Raw reads were quality-filtered to obtain clean data, followed by assembly using SOAPdenovo, SPAdes, and ABySS. The resulting assemblies were integrated using CISA, gaps were closed, and low-depth regions and short fragments were removed to obtain a high-quality draft genome.

Protein-coding genes were predicted using Prodigal v2.6.3 and GeneMarkS v4.17. Non-coding RNAs, including tRNAs and rRNAs, were identified using tRNAscan-SE v2.0 and Infernal v1.1.3 against the Rfam database. Repetitive sequences were identified using RepeatMasker v4.0.5. CRISPR elements, prophages, and genomic islands were predicted using CRT v1.2, PhiSpy v2.3, and IslandPath-DIMOB v0.2, respectively. Secondary metabolite biosynthetic gene clusters were identified using antiSMASH v5.0. Functional annotation was performed by aligning predicted protein sequences against public databases, including Nr, Swiss-Prot, TrEMBL, Pfam, eggNOG, GO, KEGG, CARD, VFDB, CAZy, TCDB, and PHI-base, using BLAST with an E-value threshold of 1e^−5^. Only hits with ≥40% coverage and ≥40% identity were retained for final annotation.

### Phylogenetic and plasmid analysis

SNPs were identified and extracted from all clinical isolates using MUMmer 3.23, and the resulting SNPs were concatenated for downstream analysis. A phylogenetic tree was constructed from concatenated SNP sequences using FastTree. Maximum likelihood phylogenetic analysis was also performed based on the single-nucleotide polymorphism (SNP) matrix using RAxML software (version 8.2.12). The nucleotide substitution model was the general time-reversible model with gamma distribution (GTRGAMMA) to account for rate heterogeneity among different sites. To assess the robustness of the phylogenetic tree topology, 1,000 rapid bootstrap replicates were performed. A phylogenetic tree was constructed based on SNPs. Genomic cluster analysis of *Corynebacterium* isolates worldwide was conducted, and a separate cluster analysis of the strains in this study was performed simultaneously. To investigate antimicrobial resistance and virulence, the GeneMarkS program was used to retrieve the related coding sequences, and Virulence Factors of Pathogenic Bacteria (VFDB) and Comprehensive Antibiotic Resistance Database (CARD) were used to predict pathogenicity and antimicrobial resistance. The highest-scoring alignments with ≥40% identity and ≥40% coverage were retained for gene annotation.

Finally, four bloodstream *C. striatum* isolates from blood infections were selected from 12 clinical strains based on combinations of four virulence gene profiles. The assembled genomes were aligned and analyzed against reference genomes in the National Center for Biotechnology Information (NCBI) database to elucidate the genetic characteristics and evolutionary relationships of these clinical isolates, using the classical *C. diphtheriae* plasmid CD1009 (NC_CP091096.1) as the reference. The NCBI plasmid database (ftp.ncbi.nlm.nih.gov//refseq/release/plasmid) was downloaded to construct a local database, and the BLAST-2.2.18 software (ftp.ncbi.nlm.nih.gov/blast/executables/blast+/LATEST) was used to align the assembled sequences against nucleotide sequences in the local plasmid database. Sequences with alignment coverage >20% were identified as potential homologous plasmids. In addition, insertion sequences (ISs) were identified by aligning genome sequences against the ISfinder database (https://www-is.biotoul.fr/blast.php)using BLAST software. Alignments were performed with a minimum threshold of 80% sequence coverage and 80% nucleotide identity, and only hits meeting both criteria were retained as valid insertion sequences.

**Fig 5:**
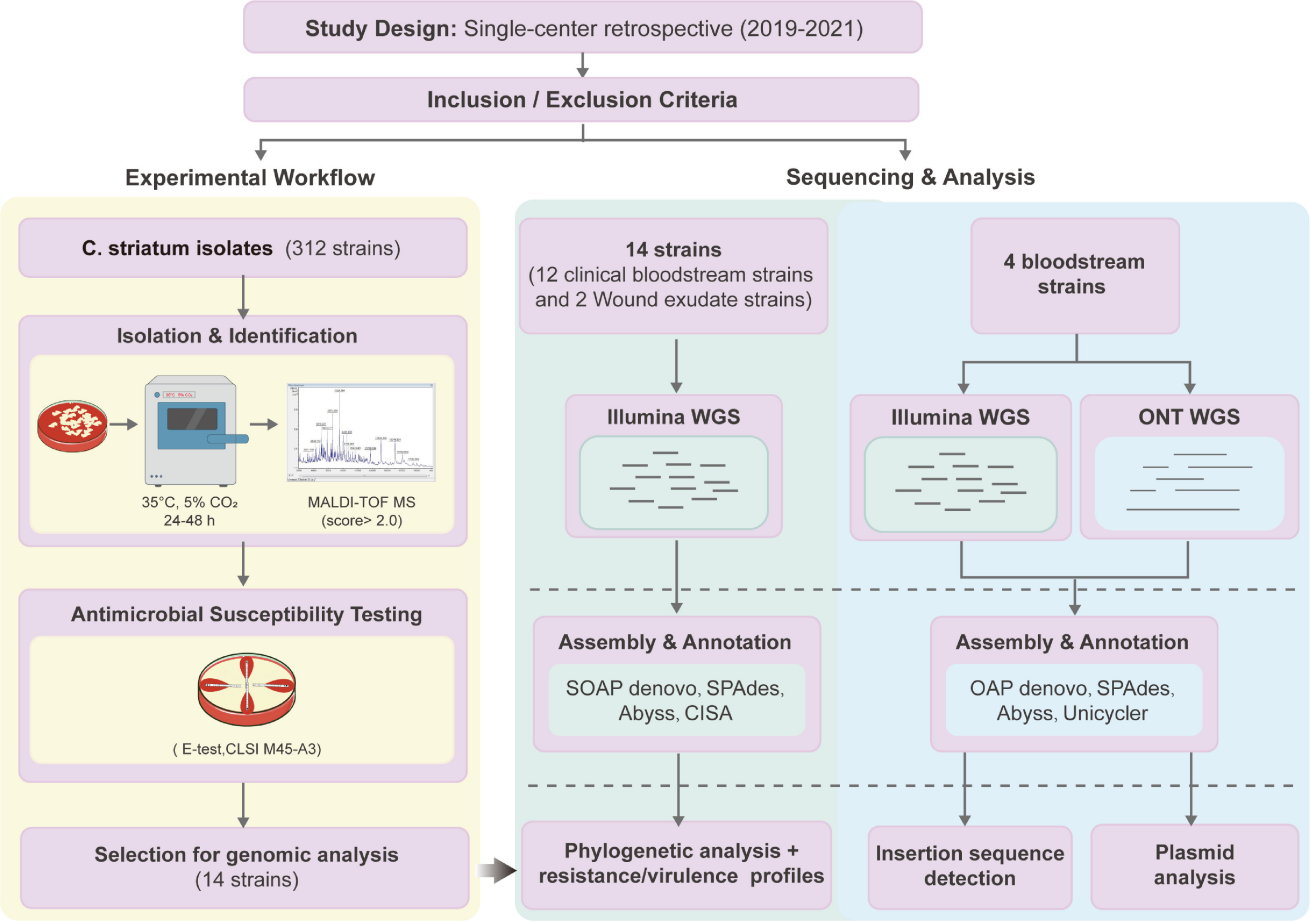
Experimental workflow for *C.striatum* genomic and phenotypic analysis. A total of 312 clinical *C. striatum* isolates were phenotypically characterized through isolation, identification, and antimicrobial susceptibility testing. Fourteen strains (12 bloodstream, 2 pus) underwent Illumina whole-genome sequencing, assembly, and phylogenetic analysis. Four bloodstream strains were additionally sequenced using Oxford Nanopore Technologies long-read sequencing for hybrid assembly, complete genome generation, plasmid analysis, and insertion sequence detection, enabling profiling of resistance, virulence, and mobile genetic elements.

Following Nanopore sequencing, based on the results of *in silico* gene prediction and functional annotation of key genetic features, a custom Perl script was developed to generate circular plasmid maps. This script used the Graphics module to parse GenBank annotation files and generate complete plasmid maps based on genomic coordinates and transcriptional directions of annotated genes. To facilitate comparative genomic analysis, the reference plasmid most closely related to the target plasmid was retrieved from the NCBI Plasmid Database based on sequence similarity. Both the target plasmid and its selected reference were uploaded to the Proksee web server (https://proksee.ca/) to generate circular comparative plasmid maps for visualization of gene content, synteny, and structural variation (Fig. 5).

### Statistical analysis

Descriptive statistics were used to characterize the study population and summarize compositional ratios. Categorical variables were expressed as frequencies and percentages to present distributions across categories. All statistical analyses were performed using IBM SPSS Statistics for Windows, Version 28.0 (IBM Corp., Armonk, NY, USA). Only descriptive statistical analyses were performed, and no inferential tests were applied.

## Data Availability

All data generated or analyzed during this study are included in this published article. The Nanopore sequencing data have been deposited in the database under the BioProject accession number PRJNA1371382.
